# Identifying the Added Value and Requirements of Telerehabilitation in Home-based Geriatric Rehabilitation: An Exploratory Qualitative Study

**DOI:** 10.5195/ijt.2024.6681

**Published:** 2025-01-15

**Authors:** Chris J. Gamble, Eleonore van Dam van Isselt, Sandra M.G. Zwakhalen, Noah Adams, Dennis P.H.G. Roekx, Jolanda C.M. van Haastregt

**Affiliations:** 1 Department of Health Services Research, Faculty of Health Medicine and Life Sciences, CAPHRI Care and Public Health Research Institute, Maastricht, the Netherlands; 2 Stichting Valkenhof, Valkenswaard, the Netherlands; 3 Department of Public Health and Primary Care, Leiden University Medical Centre, Leiden, the Netherlands

**Keywords:** Home-Based, Geriatric Rehabilitation, Telerehabilitation

## Abstract

Geriatric rehabilitation (GR) facilities are turning to innovative tools such as telerehabilitation to support home-based treatment due to challenges with population ageing, staff shortages, and mounting budgetary pressures. This study identified the potential added value as well as the requirements of using telerehabilitation in home-based GR, according to stakeholders and potential end-users. This exploratory qualitative study design conducted semi-structured interviews among nineteen care professionals, three patients and one informal caregiver. The qualitative data from these interviews were transcribed verbatim and analysed with inductive content analysis. Interviewees indicated multiple added values and requirements for telerehabilitation in home-based GR. Overall, there was great emphasis on blended care implementation, in which telerehabilitation is used in conjunction with in-person care. It is recommended to use the present findings towards developing and implementing a telerehabilitation intervention in home-based GR and assess its feasibility and usability.

Population aging is a worldwide phenomenon (World Health Organization, 2018), resulting in an increased prevalence of chronic conditions and multimorbidity (van Oostrom et al., 2014; Yarnall et al., 2017). Consequently, the need for geriatric rehabilitation (GR) is likely to rise for the coming decades (Achterberg et al., 2019). GR is defined as “*a multidimensional approach of diagnostic and therapeutic interventions, the purpose of which is to optimize functional capacity, promote activity and preserve functional reserve and social participation in older people with disabling impairments”* (Grund et al., 2020, p. 234). Evidence indicates that GR can improve functional outcomes while reducing mortality and preventing nursing home admissions (Bachmann et al., 2010; Holstege et al., 2017). While the demand for GR is rising, shortages of trained personnel and mounting budgetary pressures are increasingly straining the quality, accessibility, and affordability of GR.

In an attempt to provide high-quality GR in a cost-effective manner, GR professionals and policymakers may aim to substitute part or all of the inpatient phase with a *home-based* care trajectory, with care provided either at the patient's home or in an outpatient facility (Achterberg et al., 2019). In a recent DELPHI study on the organization of GR, consensus was reached that GR should preferably be provided in a home-based setting (van Balen et al., 2019). Similar to regular GR, evidence on home-based GR indicates improvements of functional performance and quality of life, while decreasing readmission rates, hospitalization, and rate of falls (Beswick et al., 2008; Crotty et al., 2002; Fox et al., 2013; Kiel et al., 2019; Langhorne et al., 2005; Preitschopf et al., 2023). With the majority of older adults preferring to remain in their home (Kaplan, 2022), home-based GR fits seamlessly with the *“Aging in Place”* policy, which recognises the value of delivering rehabilitation services within familiar environments to enhance patient comfort through a holistic approach (Krabbe-Alkemade et al., 2020).

Despite the potential benefits of home-based GR and the desire to increase its utilization, implementation in daily practice is proving difficult. Some notable barriers to implementation include the burden of travel time and lacking self-management support for patients and/or informal caregivers in the home environment (Prins et al., 2023). Innovative information and communication technologies (ICT) like telerehabilitation to support GR treatment remotely could facilitate these developments. Telerehabilitation, defined as “*the delivery of rehabilitation interventions to patients at a distance using ICT*” (Laver et al., 2020, # CD010255), allows for substituting the traditional in-person approach for rehabilitation to be provided at any place and any time, guided remotely by healthcare professionals (Reeder et al., 2016). While telerehabilitation is mainly praised for removing the need for travel time and lowering the care accessibility threshold (Haleem et al., 2021; Tenforde et al., 2017), additional beneficial features have emerged over time. These features include the possibility to better integrate patient skills into daily life (van Egmond et al., 2018), and to facilitate transition of care between healthcare settings by enhanced communication and information sharing when using digital patient records (Oh-Park et al., 2021). Thus, the use of telerehabilitation in GR could be advantageous and may lead to positive health-related outcomes (Kraaijkamp et al., 2021). Subsequently, telerehabilitation potentially plays a key role in solving the staff, time, and budgetary challenges faced by GR institutions.

Currently, a number of reviews have found favourable results for the effectiveness of telerehabilitation in community-dwelling older adults with rehabilitation needs (Ashe et al., 2018; Batsis et al., 2019; Gamble et al., 2024; Reeder et al., 2016; Saito & Izawa, 2021; Velayati et al., 2020; Wicks et al., 2023). When compared to usual, in-person care, similar results on patient- and health related outcomes such as functional performance, quality of life, and physical functioning have been found. Although these findings are not specific to GR, they do offer a glimpse into the potential of this approach for home-based GR. Still, successful implementation of telerehabilitation in home-based GR is complex, and more insight is needed on its feasibility and usability (Kraaijkamp et al., 2021). Since there is little knowledge on the use of telerehabilitation in the specific domain of home-based GR, it is imperative to analyse *when* and *how* telerehabilitation can be of added value (Kip et al., 2019a). Values specify what users want to achieve or improve when working with a technology such as telerehabilitation (Kip et al., 2019a). It can be challenging to assess the values of everyone involved in the rehabilitation process, especially from a frail population who might have difficulties in formulating their thoughts and opinions (Kip et al., 2019b). Thus, when considering the application of telerehabilitation in home-based GR, researchers should focus on user acceptance and engagement to ensure a good fit with their needs and preferences.

At present, to our knowledge no studies have investigated how telerehabilitation interventions should be implemented in the complex setting of home-based GR. Moreover, the added value and requirements of implementing telerehabilitation in home-based GR are unknown. Therefore, this study aimed to identify the potential added value as well as the requirements of using telerehabilitation in home-based GR, according to stakeholders and potential end-users.

## Methods

### Design

The current research employed an exploratory qualitative study design and was based at Stichting Valkenhof, a multidisciplinary care facility with GR based in Valkenswaard, The Netherlands.

### Study Population

Based on participatory development design, the study population consisted of the (proposed) telerehabilitation system end-users, as well as other stakeholders involved in policy and management in GR facilities (Kukafka et al., 2003). A wide range of care professionals, as well as patients and informal caregivers, were eligible for inclusion.

Recruitment was done by purposive sampling by the primary author (CG) in order to select cases that optimally contribute to data collection and the objective of the study. Recruitment took place in February through May 2023 (Polit, 2019). To be eligible for inclusion, care professionals had to (1) be employed for Stichting Valkenhof for a minimum of six months preceding the start of the study, (2) be involved in any way with home-based GR or primary care for recently discharged GR patients, (3) be able to speak Dutch or English, and (4) provide informed consent. For patients and informal caregivers, the eligibility for inclusion was determined by (1) received home-based GR by a maximum of twelve months preceding the start of the study, (2) the ability to perform a semi-structured interview assessed by the currently involved healthcare professionals, (3) not receiving palliative care, (4) the ability to speak Dutch or English, and (5) legally competent to provide written informed consent. Information on the methodology and topic of the study was provided via an information letter. Participants were given a reflection period of five working days and subsequently asked to provide written informed consent. Participants could withdraw at any given time.

### Data Collection

Semi-structured interviews were used to gather data from participants. By doing so, it was assured that detailed answers could be given on key research questions, while enabling probing questions if necessary to gain more insight into a participant's thoughts on a subject. The interview scheme and topic list was partly based on the qualitative approach used by Kip et al. (2019a). The interviews started with a brief introduction, with these subsequent elements: a brief explanation on the nature of the interview and the study; a brief explanation on the term “telerehabilitation;” a statement on interview data collection and storage; a confirmation of willingness to participate; and finally, questions on demographics, including age, gender, profession and working experience. The primary themes of the topic list for the interview were to (1) determine the current application or experience with home-based GR and telerehabilitation, (2) determine the added value of telerehabilitation in home-based GR and (3) determine the requirements of using telerehabilitation in home-based GR. The full topic list is included in Appendix A in the original Dutch language, with a translated version found in Appendix B. A more straightforward version with less technical terms was used for patients and the informal caregiver.

The interviews were held in-person or online using video conferencing software, depending on the preference and schedule of the participant. For all patients and the informal caregiver, all interviews were held in-person in their home, in order to minimalize their burden. The interviews had a scheduled duration of 30 minutes. Pilot interviews were held with two physiotherapists and one patient to confirm the scheduled duration and provide feedback on the contents, with minor adjustments made accordingly. All interviews were recorded with the permission of the participants. A member check was performed after the interview to strengthen credibility of the data, by providing each participant with a summary of the coded interview (Koelsch, 2013). The Medical Research Involving Human Subjects Act in the Netherlands does not apply to this study and therefore official approval of this study by a Medical Ethics Committee was not required. However, ethical approval was provided by the Research Ethics Committee of Maastricht University (Faculty of Health, Medicine and Life Sciences).

### Data Analysis

All audio recordings from the interviews were transcribed verbatim using Microsoft Word by two researchers (DR and NA). All data was pseudonymized to ensure no data can be linked to any participant. Subsequently, the transcripts were coded independently using Atlas.ti version 9.0. Analysis was performed using an inductive content analysis (Moser & Korstjens, 2018). Via the inductive content analysis, interview data was broken down and attached to specific headings. By comparing, grouping, and sub-dividing sets of interview data with specific headings, subthemes containing units of similar content were formed. The subthemes with similar content were then matched with the main themes that were pre-determined by deductive coding.

## Results

Twenty-three interviews with nineteen care professionals experienced in GR, three former GR patients, and one informal caregiver, were conducted in May and June 2023 by two researchers (DR and NA). The characteristics of the participants are shown in Table 1. Professions included were a physician (specialised in care for older persons), GR care coordinators, GR nurse, care consultants, GR manager and a range of GR therapists (physiotherapist, occupational therapist, speech therapist, and dieticians). All participants were female, except for the GR manager and two patients. The care professionals were aged between 26–61 years old with a mean age of 39 years and had an average working experience in the field of GR of 11 years. The three patients and informal caregiver were aged between 67 and 82 years old. All interviews were conducted in Dutch and the average duration was 34 minutes. All three patients received home-based or outpatient primary care following inpatient GR.

**Table 1.** Demographics of Study Participants

Healthcare professional	Occupation	Gender/Age (range)	Working experience (years)
**CP 1**	Physiotherapist	F, <30	5–10
**CP 2**	Physiotherapist	F, 40–50	15>
**CP 3**	Physiotherapist	F, <30	5–10
**CP 4**	Physiotherapist	F, <30	<5
**CP 5**	Occupational therapist	F, <30	<5
**CP 6**	Occupational therapist	F, 30–40	15>
**CP 7**	Occupational therapist	F, 30–40	5–10
**CP 8**	Occupational therapist	F, 30–40	5–10
**CP 9**	Speech therapist	F, 30–40	<5
**CP 10**	Speech therapist	F, 30–40	5–10
**CP 11**	Dietician	F, 30–40	5–10
**CP 12**	Dietician	F, 60>	15>
**CP 13**	GR Care Coordinator	F, 30–40	<5
**CP 14**	GR Care Coordinator	F, 40–50	15>
**CP 15**	Care Consultants	F, 60>	15>
**CP 16**	Care Consultants	F, 40–50	15>
**CP 17**	GR Nurse	F, 60>	15>
**CP 18**	GR Manager	M, 30–40	<5
**CP 19**	Physician (specialized in care for older persons)	F, 50–60	15>

**Patient**		**Gender/Age**	**Level of education**
**P 1**	-	F, 67	Secondary vocational
**P 2**	-	M, 76	Higher professional
**P 3**	-	M, 77	University
**IC 1**	-	F, 82	Primary

*Note*. CP, care professional; P, patient; IC, informal caregiver; F, female; M, male; GR, geriatric rehabilitation

### Current Application and Experience with Home-based GR and Telerehabilitation

To determine the potential added value and requirements of telerehabilitation in home-based GR, the interviewees were first asked about the current application and experience with home-based GR, as well as the current application and experience with telerehabilitation, as practised within Stichting Valkenhof. The insights provided were not directly related to the study aims and are therefore presented in Appendix C, Table 4.

### Telerehabilitation in Home-based GR

For telerehabilitation in home-based GR, the main themes, identified sub-themes and their definitions, are provided in Table 2. Telerehabilitation in home-based GR was categorized in potential added value and potential weaknesses.

**Table 2.** Potential Added Value and Weaknesses of Telerehabilitation in Home-based GR

Main themes/sub-themes	Definition of theme	Codes^a^	CP^b^	P/IC^c^
**Potential added value of telerehabilitation in home-based GR**				
- **Absence of travel time**	- Telerehabilitation does not require transportation	**14**	**12**	2
- **Home rehabilitation**	- Telerehabilitation may increase patient comfort and well-being due to rehabilitating at home.	**10**	**7**	**3**
- **Improved independent functioning**	- Telerehabilitation may lead to improved self-management skills and independent functioning.	**9**	**7**	**2**
- **Mnemonic**	- Telerehabilitation may lead to exercising more often.	**5**	**5**	-
- **Increased contact-time**	- Telerehabilitation may lead to more frequent interaction between professional and patient.	**5**	**5**	-
- **Cost-effectiveness**	- Due to a combination of several factors, telerehabilitation may be more cost-effective.	**14**	**12**	**2**
- **Exercise visualisation**	- Visualisation by videos or videoconferencing may be beneficial for certain rehabilitation elements.	**7**	**6**	**1**
- **Innovative care**	- Implementing telerehabilitation in home-based GR would be innovative, distinguishing Valkenhof from their competitors.	**3**	**3**	-
- **Blended care possibilities**	- Telerehabilitation may negate most weaknesses when used in addition to in-person contact.	**11**	**8**	**3**
**Potential weakness of telerehabilitiation in home-based GR**				
- **Social isolation**	- In rehabilitation with older adults, face-to-face contact may sometimes be preferred or necessary.	**15**	**13**	**2**
- **Guidance difficulties**	- Some rehabilitation components may require or benefit from in-person guidance.	**10**	**10**	-
- **Burden**	- Telerehabilitation may be burdensome for some older adults or their informal caregivers.	**13**	**10**	**3**
- **Investment requirements**	- Incorporating telerehabilitation may require monetary investments for the care organisation.	**2**	**2**	-

*Note*. *CP*, care professional; *P*, patient; *IC*, informal caregiver*; GR*, geriatric rehabilitation;

a The total number of times a code was mentioned in all interviews;

b The number of different therapists that mentioned a code;

c The number of patients or informal caregiver that mentioned a code

#### Added Value of Telerehabilitation in Home-based GR

All identified sub-themes regarding the potential added value of telerehabilitation in home-based GR is provided in Table 2. The most mentioned potential added value of telerehabilitation is the time gained, or rather not lost, by traveling either to the patients’ home or to the care organisation. In addition, care professionals and patients expressed that telerehabilitation in home-based GR could be more cost-effective, due to reduced travel time and more potential for efficient scheduling. Moreover, with patients receiving home-based GR in part by telerehabilitation, this can lead to more efficient use of in-patient treatment capacity. Care Professional 6 noted that:

Quote: *“If I make a home visit for an evaluation, it will take me at least an hour; travel time, being there, maybe have a small chat. This is for someone who does not even live far away. If we were to do that digitally, you could see maybe four people in an hour. So that would certainly be cost-effective.”* (CP 6)

Furthermore, telerehabilitation has the potential benefit of allowing older adults to rehabilitate at home, in their own environment, which may increase patient well-being, independent functioning and social participation. Lastly, telerehabilitation may serve as a mnemonic, potentially increasing home rehabilitation frequency, as the following quotes illustrate:

Quote: *“We notice when people are admitted to GR, it has an impact on them. Many have already been in hospital for some time, and then they come here. They are away from home for a long time and that can have adverse effects on a patient. When you are at home, you are in your own environment, I can imagine it is advantageous for the well-being of the patient.”* (CP 15)

Quote: *“I think the advantage is that they can spend more time on their rehabilitation at home, even when we are not there. When we come by, patients think about their rehabilitation, the exercises are done, but if we are not there, they often forget to do their exercises. Telerehabilitation could stimulate patients to do their homework exercises and serve as a reminder for them.”* (CP 5)

#### Weaknesses of Telerehabilitation in Home-based GR

All identified sub-themes regarding the potential weaknesses of telerehabilitation in home-based GR is provided in Table 2. Social isolation and an increased patient or informal caregiver burden were the two most mentioned potential weaknesses of telerehabilitation in home-based GR. Both issues, however, were expressed under the assumption that telerehabilitation would be used for most, if not all, appointments in home-based GR. While this may or may not necessarily be the case in practise, the following quote illustrates one of the potential issues that may arise:

Quote: *“Older adults are, most of the times, genuinely very happy when they see someone. They are often lonely people who either live alone at home or in a room somewhere, and that personal contact is of great value. […] At a certain point, a therapist becomes a bit of a confidant. If that contact, in person, is lost, then it really is a great loss, a decline I would say. That really is a disadvantage, loosing that social aspect of our profession.”* (CP 1)

Telerehabilitation implies there is a physical distance between the care professional and patient, as guidance is provided digitally. Some professions, like physiotherapists who are accustomed to providing physical guidance to frail older adults, may be hesitant to provide guidance at a distance. While safety issues could potentially be a concern, the difficulties of providing guidance at a distance go beyond that. According to the care professionals, certain skills training or observations can only be done in person.

Quote: *“People with aphasia, with them I can easily see us working digitally; or play videos of the exercises for them on a screen. However, sometimes we need to study the breathing or swallowing of a patient, and I'm not sure this can be done by telerehabilitation. Not only because it can be hard to see on a screen, but because I need to manually check it.”* (CP 10)

### Implementing Telerehabilitation in Home-based GR

For implementing telerehabilitation in home-based GR, the main themes, identified sub-themes and their definitions are provided in Table 3. The implementation of telerehabilitation in home-based GR was categorized as to requirements, facilitating factors, and inhibiting factors.

**Table 3.** Requirements, Facilitating Factors and Inhibiting Factors for Telerehabilitation in Home-based GR

Main themes/sub-themes	Definition of theme	Codes^a^	CP^b^	P/IC^c^
**Requirements of telerehabilitation in home**				
- **Clear in- and exclusion criteria**	- Telerehabilitation may benefit from clear in- and exclusion criteria to create the best fit for the patient.	**13**	**13**	**-**
- **IT infrastructure**	- For a smooth integration in current work processes, the IT infrastructure of telerehabilitation should be arranged.	**10**	**8**	**2**
- **Keep it simple**	- Telerehabilitation should be kept as simple as possible for improved feasibility.	**8**	**7**	**1**
- **Training sessions**	- Training in the use of telerehabilitation	**17**	**13**	**4**
- **Blended care**	- Combining telerehabilitation with in-person care may be best to achieve all rehabilitation goals.	**11**	**8**	**3**
**Facilitating factors for telerehabilitation in home-based GR**				
- **In-patient training**	- Introducing and training with the telerehabilitation application during inpatient stay.	**11**	**11**	**-**
- **Prior experience**	- Any previous experience with IT or digital devices was deemed to be beneficial to use telerehabilitation.	**11**	**11**	**-**
- **Device loan service**	- Enable the use of telerehabilitation by having a device loan service.	**8**	**8**	**-**
- **Informal caregiver support**	- Assistance from an informal caregiver may be beneficial when using telerehabilitation	**4**	**4**	**-**
**Inhibiting factors for telerehabilitation in home-based GR**				
- **Usability issues**	- Physical or cognitive impairments are quite common among GR patients, which may impede the use of telerehabilitation.	**16**	**14**	**2**
- **Device related issues**	- Internet connection or device settings may need to be arranged for the patient.	**16**	**14**	**2**
- **Unsafe home environment**	- As with home-based GR in general, the home environment may be unsuited for telerehabilitation.	**4**	**4**	**-**

*Note*. *CP*, care professional; *P*, patient; *IC*, informal caregiver*; GR*, geriatric rehabilitation;

a The total number of times a code was mentioned in all interviews;

b The number of different therapists that mentioned a code;

c The number of patients or informal caregiver that mentioned a code

#### Requirements of Telerehabilitation in Home-based GR

All identified sub-themes regarding the requirements of telerehabilitation in home-based GR are provided in Table 3. The two primary requirements indicated by care professionals, patients, and the informal caregiver were familiarity with the telerehabilitation application or device by training, and a smooth IT infrastructure. A smooth IT infrastructure includes conditions such as: who provides the telerehabilitation device; when the device is provided; how the internet connection is established; how the application settings are arranged; how a telerehabilitation e-consultation is structured; and more. Furthermore, training sessions should be held to familiarise patients and informal caregivers with the device, how to use it, the benefits of such use, as well as to inform care professionals of all the possibilities of telerehabilitation. The following two quotes relate to these needs:

Quote: *“[…] Of course, treatment by telerehabilitation requires a different approach than in-person. How do you explain things well? The way you approach things physically might not come across so well online, so that might require some training.”* (CP 19)

Quote: *“[…] Right now, I don't think we are set up for it (…for telerehabilitation) to do things digitally, because everything is still focused on paper. This should be addressed.”.* (CP 9)

Another commonly mentioned prerequisite for telerehabilitation was to have clear inclusion and exclusion criteria. Since age-related impairments are very common in GR, some telerehabilitation applications or devices might be a good fit for some patients, but not work for others. Moreover, it was noted for telerehabilitation to ‘keep it simple’. Telerehabilitation applications or devices should be clear, easy to use and able to be operated by older adults with limited IT skills.

Quote: *“The easier the app or guide is, the easier patients will reach out for them, right? Because if it is too complicated, they will be put aside and not be used.”* (CP 17)

Lastly, while not necessarily a prerequisite, most of the care professionals and patients did emphasize that the use of telerehabilitation should be combined with in-person care. Having home-based GR exclusively by telerehabilitation was not deemed to be feasible, while blended care might achieve in bringing the best of both worlds together.

Quote: *“I think that telerehabilitation would definitely offer advantages, but we should not forget that we are working with a frail population that still needs in-person contact, so this (…telerehabilitation) would be supplemental. We should work with this; as an addition to the current rehabilitation.* (CP 18)

#### Facilitating Factors for Telerehabilitation in Home-based GR

All identified sub-themes regarding the facilitating factors for telerehabilitation in home-based GR are provided in Table 3. First, introducing the telerehabilitation application or device while still in the in-patient setting of GR would be beneficial. This enabled patients to become familiar with the device with trial-and-error practise during the in-patient stay. Care professionals also pointed out how having a loan service for telerehabilitation appliances could facilitate the uptake of telerehabilitation. The quote below asserts this point. Doing so could greatly reduce the burden on the patient and informal caregiver, while contributing to a smooth IT infrastructure that is compliant with current work processes. The last facilitating factor for telerehabilitation in home-based GR mentioned by care professionals was the possibility of informal caregiver support.

Quote: *“I think, as an organization, you should facilitate this (…telerehabilitation), by lending out the device for telerehabilitation temporarily. The organization should give the device on loan, this way we can control the settings and make sure it works.”* (CP 16)

#### Inhibiting Factors for Telerehabilitation in Home-based GR

All identified sub-themes regarding the inhibiting factors for telerehabilitation in home-based GR are provided in Table 3. Usability concerns due to cognitive or sensorimotor issues were mentioned by most interviewees. These concerns were related to the target population, with many if not all older adults in GR having at least some decline in cognitive or sensorimotor control or functioning, potentially prohibiting the use of telerehabilitation. Device-related issues were also commonly mentioned. Yet, these could be partly countered by having a loan service and an accessible IT infrastructure.

Quote: *“If I, let's say, have a patient in his sixties who only broke his hip, is here for two weeks and recovers enough to go home, then I think there is no problem (…to use telerehabilitation in home-based GR). But that is a very, very small part of the patients we see in GR now. The vast majority either have some sort of cognitive problem, or a combination with physical issues or other comorbidities that make the use of telerehabilitation difficult”.* (CP 6)

## Discussion

This study aimed to identify the potential added value of telerehabilitation in home-based GR, as well as the requirements when using telerehabilitation in home-based GR. We completed twenty-three interviews with healthcare professionals, patients, and an informal caregiver experienced with GR. The study was based in the care organization Stichting Valkenhof.

To determine the potential added value and requirements of telerehabilitation in home-based GR, interviewees were first asked about their perspectives on the current strengths and weaknesses of home-based GR. Most of the care professionals, however, mentioned that the use of home-based GR within Stichting Valkenhof was limited. In the opinion of the care professionals, this was due to issues with regulations and the financial reimbursement structure, as well as dealing with a frail target population. Several care professionals expressed the desire to increase the use of home-based GR. Home-based GR was believed to be of value particularly after a brief in-patient stay. The desire of care professionals of Stichting Valkenhof to increase the use of home-based GR is in accordance with a DELPHI study with European consensus that GR should preferably be offered in an outpatient setting (van Balen et al., 2019). Moreover, van den Besselaar et al. (2021) indicated that GR providers want increased utilization of outpatient GR. With the possible addition of telerehabilitation in home-based GR, care professionals might feel empowered to take this step.

The application of telerehabilitation in home-based GR was categorized in potential added value and potential weaknesses. Most of the interviewees found the following potential added value for telerehabilitation in home-based GR: the absence of travel time; increased cost-effectiveness and the potential for more revenue for the care organization; improved patient comfort and well-being due to rehabilitating at home; improved self-management skills; and the potential for blended care. Blended care, (i.e., telerehabilitation and in-person therapy are combined), may provide several advantages while negating most of the perceived weaknesses.

Most of the interviewees put forth three potential weaknesses for telerehabilitation in home-based GR: the potential for social isolation; increased patient and/or caregiver burden; and the potential for guidance difficulties for rehabilitation components that require in-person supervision.

In addition, the implementation of telerehabilitation in home-based GR was categorized into requirements, facilitating factors, and inhibiting factors. Important requirements of telerehabilitation in home-based GR were training sessions for familiarity with the telerehabilitation application or device; using clear inclusion and exclusion criteria; a thoughtful IT infrastructure for a smooth integration in current work processes; and the notion for telerehabilitation to ‘keep it simple’. The main perceived facilitating factors were the early introduction of telerehabilitation training during in the inpatient phase; having previous experience with digital media; and offering a device loan service to supply patients with all telerehabilitation necessities. Several inhibiting factors for telerehabilitation in home-based GR were mentioned, namely: the potential for usability issues due to physical or cognitive impairments in the target population; and that device related issues might prohibit a seamless telerehabilitation experience.

The potential added value of telerehabilitation in home-based GR as specified in the current study, is consistent with current telerehabilitation research in older adults. An obvious advantage of telerehabilitation is the absence of travel time (Wicks et al., 2023). While investigating home-based telerehabilitation in an older adult population, Crotty et al. (2014) reported that the travel time of care professionals reduced by up to 50%, with time savings allocated to provide care to more people. In doing so, telerehabilitation could meet the growing demand for rehabilitation services due to an ageing population, while remaining cost-effective (Snoswell et al., 2020). Moreover, Crotty et al. (2014) found telerehabilitation to improve accessibility and increase direct contact-time with patients, thus potentially easing the transition from in-patient to home-based care. In addition, by utilizing educational sessions for patients to improve the familiarity with telerehabilitation (Wicks et al., 2023), the need for present informal caregivers might be diminished. These findings suggest that the potential added value of telerehabilitation might neutralise several of the barriers for adoption of home-based GR as experienced by care professionals (Appendix C, Table 4).

Blended care, in which telerehabilitation and in-person care are combined, was the most mentioned positive approach to home-based GR. This form of hybrid care is likely to be the future of geriatric telerehabilitation, a viewpoint shared by many studies in this field (Haleem et al., 2021; Marzuca-Nassr et al., 2022; Oh-Park et al., 2021). In fact, in a recent review on eHealth. Kraaijkamp et al. (2021) showed that blended care is more likely to be feasible for older adults receiving GR, especially when the eHealth interventions were kept simple.

Both the current results and those reported by Jørgensen et al. (2021) support the use of an easily used, uncomplicated telerehabilitation platform. Similar to the present study, Jørgensen et al. (2021) encountered several facilitating and inhibiting factors for telerehabilitation in home-based GR. Primarily, these factors related to the desire for familiarity with the telerehabilitation device by training; access to a mature IT platform; and being mindful of usability issues in a frail population recovering from acute illness. Jørgensen et al. (2021) therefore concluded that it may not be advisable to immediately start telerehabilitation after discharge home from hospital.

Usability issues have been a primary concern for studies investigating telerehabilitation in an older adult population (Wildenbos et al., 2018). However, a systematic review by Wicks et al. (2023) found strong adherence when older adult patients used telerehabilitation instead of in-person rehabilitation. Wicks et al. (2023) argued that usability issues might actually work in favor of telerehabilitation, which may remove barriers when accessing in-person rehabilitation such as the need for transportation, physical access, travel fatigue and mobility issues.

### Strength and Limitations

This study has several limitations. First, it is possible that the interviewees answered in a socially desirable way, which might have decreased the credibility of the data. To minimize this potential bias, all participants were reminded that their data would be processed anonymously. In addition, a member check was performed to strengthen the credibility of the data. Second, due to the exploratory study design in a single care facility, it is possible that the perspective shared by care professionals and patients in this study differ from the opinion of GR professionals in general. Furthermore, despite including a wide variety of care professionals, patients and an informal caregiver, no ICT staff was interviewed during this study, potentially excluding relevant viewpoints. Lastly, several interviewees had limited experience with home-based GR and telerehabilitation. Their viewpoints were therefore based on perceived added value and requirements of telerehabilitation in home-based GR. While this might have influenced the results, it could also be seen as a strength since their viewpoints are important to consider when implementing telerehabilitation in home-based GR. Another strength of the study is the qualitative approach within a wide variety of care professionals, which allowed for relatively rich information to be obtained in a short period.

### Recommendations

It is recommended to use the present findings towards developing and implementing a telerehabilitation intervention designed to assist in home-based GR. Of note should be to apply a blended form of easily used telerehabilitation, with attention to the IT infrastructure and early training sessions to increase device familiarity. The feasibility of telerehabilitation use in home-based GR should be assessed (including adherence and acceptability) as it has not yet been established (Ossebaard et al., 2013). In many cases of e-Health development, non-adherence is the driving issue behind lacking implementation which impairs it's feasibility (Pieterse et al., 2018). Proposed reasons for non-adherence of telerehabilitation could be a mismatch between patient goals and intervention capabilities, the inability to integrate the technology into daily routines, or usability issues. Usability issues, such as motor sensory or cognitive problems, may impede the use of telerehabilitation by older adults and require telerehabilitation to be sufficiently tailored to the frail, multimorbid population found in GR (Foster & Sethares, 2014; Wildenbos et al., 2018).

## Conclusion

This study aimed to identify the potential added value and requirements of telerehabilitation in home-based GR. Despite perceived barriers, a majority of care professionals expressed the added value of telerehabilitation in home-based GR, most notably the absence of travel time and improved cost-effectiveness. Regarding the requirements, there was great emphasis on training sessions to increase device familiarity, as well as implementation of blended care when telerehabilitation is used in conjunction with in-person care. It is recommended to use the present findings towards developing and implementing a telerehabilitation intervention in home-based GR and assessing its feasibility and usability.

## Appendix A Full Interview Scheme (Original, in Dutch)

**Topiclijst semi-gestructureerd interview**

**BEHANDELAREN/OVERIGE FUNCTIES**

1. **Introductie:**
  - *Voorstellen interviewer.*
  - *Korte uitleg over de studie en de vragen die aan bod komen.*
  Om geriatrische revalidatie toegankelijk en betaalbaar te houden wordt er door Valkenhof samen met de Universiteit Maastricht onderzoek gedaan naar nieuwe manieren om deze zorg vorm te geven. Wij willen graag onderzoeken of telerevalidatie een zinvolle aanvulling kan zijn voor mensen die thuis wonen en ambulante geriatrische revalidatie ontvangen. Onder ambulante GRZ verstaan we in deze studie revalidatiezorg die in de thuissituatie of poliklinisch wordt aangeboden.
  - *De term ‘telerevalidatie’ uitleggen, navragen of het duidelijk is.*
  In dit onderzoek bedoelen we met telerevalidatie revalidatiezorg die op afstand wordt aangeboden met behulp van technologie. Een voorbeeld hiervan is een behandelaar die een client begeleidt bij het thuis doen van oefeningen, via videobellen of telefonisch contact. Is dit duidelijk voor u?
  - *Toestemming vragen voor deelname en opname van het gesprek*
  - *Uitleg over wat er met de gegevens wordt gedaan*
  Graag wil ik even toelichten wat er met de gegevens van dit interview gebeurt. Het interview wordt opgenomen met een telefoon. Is het akkoord voor u dat wij het gesprek opnemen? De opname van het interview wordt uitgeschreven en geanalyseerd. Vervolgens wordt een samenvatting van het gesprek gemaakt die we u vervolgens toesturen om na te gaan of we uw antwoorden goed hebben geïnterpreteerd. Als u nog aanpassingen of aanvullingen hebt kunt u dat aan ons doorgeven binnen 5 werkdagen. Uw gegevens worden vervolgens geanonimiseerd verwerkt. Dit betekent dat in de uiteindelijke beschrijving van de resultaten uw naam niet genoemd wordt en de gegevens niet herleidbaar zullen zijn, naar u als persoon.
  - *Aangeven dat het gesprek naar verwachting 30 min duurt en men altijd tussendoor kan aangeven om te pauzeren.*
  Tijdens dit interview gaan wij u vragen stellen over de huidige toepassing van ambulante geriatrische revalidatiezorg en telerevalidatie binnen Valkenhof. Wij gaan u als eerste vragen naar uw mening over de organisatie van de ambulante GRZ binnen Valkenhof. Wij gaan u vervolgens vragen stellen over de mogelijke rol en toepassing van telerevalidatie binnen de ambulante GRZ. En als laatste stellen wij u vragen over wie er belang heeft of zou kunnen hebben bij het toepassen van telerevalidatie in de ambulante GRZ (stakeholder identificatie).
2. **Achtergrondkenmerken:**
  - Bevragen/noteren kenmerken van de respondent. Dit zijn:
    – Geslacht
    – Leeftijd
    – Functie
    – Werkervaring (jaren)
3. **De huidige toepassing van ambulante GRZ en telerevalidatie binnen Valkenhof**
  - In hoeverre biedt Valkenhof ambulante GRZ? In welke vorm wordt het geboden (poliklinisch of bij de patient thuis?)
  - In hoeverre bent u zelf betrokken bij de uitvoering van ambulante GRZ?
    – In welke vorm biedt u ambulante GRZ (poliklinisch of bij de patient thuis?)
    – *Indien de persoon niet betrokken is bij ambulante GRZ, vraag dan*:
      In hoeverre bent u betrokken bij eerstelijns zorg aan GRZ patienten die naar huis zijn ontslagen?
  - In hoeverre wordt binnen uw organisatie gebruik gemaakt van telerevalidatie?
    – Waar wordt dit toegepast? GRZ-klinisch? GRZ-poliklinisch? GRZ-thuisbehandeling bij ambulante GRZ? In de eerstelijnszorg?
    – Welke vormen van telerevalidatie worden toegepast?
  - In hoeverre maakt u zelf gebruik van telerevalidatie?
    – Waar gebruikt u dit? GRZ-klinisch? GRZ-poliklinisch? GRZ-thuisbehandeling bij ambulante GRZ? In de eerstelijnszorg?
    – Van welke vormen van telerevalidatie maakt u gebruik?
      - Therapie op afstand via videobellen?
      - Het samen met de patient gebruiken van revalidatieapps, bijv. voor het doen van oefeningen?
      - Het gebruik van bewegingssensoren en op basis daarvan de patient coachen/monitoren?
      - Anders, namelijk….?
4. **Uw mening over de ambulante GRZ**
  - Wat is uw mening over de ambulante GRZ?
    – Wat gaat er volgens u goed? Kunt u voorbeelden noemen?
    – Wat gaat er volgens u minder goed? Kunt u voorbeelden noemen?
    – Kunt u aangeven wat de oorzaken zijn van de dingen die u minder goed vindt gaan?
    – Welke dingen zouden volgens u verbeterd kunnen worden in de ambulante GRZ?
  - *Indien men aan heeft gegeven dat er géén ambulante GRZ wordt geboden bij Valkenhof, onderstaande vragen stellen*:
    – Waarom wordt ambulante GRZ niet toegepast bij Valkenhof?
    – Wat vindt u ervan dat er geen ambulante GRZ wordt toegepast?
    – In hoeverre zou de ambulante GRZ volgens u moeten worden toegepast? Welke vorm zou dit dan moeten hebben (poliklinisch, behandeling thuis)
5. **De mogelijke rol van telerevalidatie binnen de ambulante GRZ**
  - *Indien men bij vraag 4 aangegeven heeft dat er bij ambulante GRZ verbeterpunten zijn, dan onderstaande vragen stellen:*
    U geeft aan dat de volgende dingen verbeterd zouden kunnen worden in de ambulante GRZ: *[noem hier de bij vraag 4 genoemde dingen]*.
    Denkt u dat telerevalidatie hierbij een rol kan spelen?
    – Zo ja, kunt u dit toelichten? Hoe zou dit er dan uit moeten zien volgens u? Wat is hierbij belangrijk?
    – Zo nee, ziet u wellicht andere mogelijkheden om het ambulante revalidatieproces te verbeteren met het gebruik van telerevalidatie?
    ▪ Zo ja, hoe zou dit er dan uit moeten zien volgens u? Benoem alle aspecten die voor u hierbij belangrijk zijn.
    ▪ Zo nee, wat is de reden dat u geen mogelijkheden ziet om het revalidatieproces te verbeteren met het gebruik van telerevalidatie?
  - *Indien men bij vraag 4 aangegeven heeft dat er wél ambulante GRZ wordt geboden maar geen verbeterpunten zijn, of dat er géén ambulante GRZ wordt geboden, dan onderstaande vragen stellen:*
    Ziet u wellicht mogelijkheden om het ambulante revalidatieproces (in het algemeen of binnen Valkenhof) te verbeteren met gebruik van telerevalidatie?
    – Zo ja, hoe zou dit er dan uit moeten zien volgens u? Benoem alle aspecten die voor u hierbij belangrijk zijn.
    – Zo nee, wat is de reden dat u geen mogelijkheden ziet om het revalidatieproces te verbeteren met het gebruik van telerevalidatie?
6. **Potentiële meerwaarde en nadelen van telerevalidatie binnen de ambulante GRZ**
  - Wat is volgens u de meerwaarde van telerevalidatie in een ambulant revalidatietraject?
    – Voor de patient?
    – Voor de naasten/mantelzorgers?
    – Voor de zorgverleners?
    – Voor de zorgorganistie?
    – Voor de doelmatigheid/efficiëntie van de zorg?
    – Voor andere zaken, namelijk:
  - In hoeverre zijn er volgens u nadelen verbonden aan het gebruik van telerevalidatie in een ambulant revalidatietraject
    – Voor de patient?
    – Voor de naasten/mantelzorgers?
    – Voor de zorgverleners?
    – Voor de zorgorganistie?
    – Voor de doelmatigheid/efficiëntie van de zorg?
    – Voor andere zaken, namelijk:
7. **Factoren die toepassing van telerevalidatie kunnen beïnvloeden**
  - Welke factoren kunnen volgens u de toepassing van telerevalidatie in een ambulant revalidatietraject bevorderen?
  - Welke factoren kunnen volgens u de toepassing van telerevalidatie in een ambulant revalidatietraject belemmeren?
  - Wat is er volgens u nodig om telerevalidatie goed te kunnen implementeren in de ambulante GRZ (binnen Valkenhof)? Zowel voor patiënt, de naasten, de zorgverleners als de organisatie?
    – Beschikbare materialen en voorzieningen?
    – Scholing medewerkers
    – Educatie patiënten en naasten
    – Vormgeving van de te gebruiken software?
    – Organisatie van de zorg?
    – Ondersteuning van de patient door naasten?
    – Declareren van telerevalidatie? Aanpassen huidige regelgeving?
    – Beschikbaarheid van zorgpaden of richtlijnen?
    – Overig?
8. **Het belang van verschillende partijen (stakeholders) bij het toepassen van telerevalidatie in de ambulante GRZ?**
  *Bepaalde mensen kunnen in verschillende mate belang hebben bij het toepassen van telerevalidatie in de ambulante GRZ. Daarom willen wij nu graag kijken naar wie er belang kan hebben bij het toepassen van telerevalidatie in de ambulante GRZ.*
  - Wie hebben er volgens u belang bij het toepassen van telerevalidatie in de ambulante GRZ?
  - Wie heeft er volgens u het meeste belang bij? Hebben bepaalde personen/partijen er meer belang bij dan andere?
    – Zo ja, wie zou er dan belangrijker zijn?
    – Wat is de redenen dat deze personen/partijen er volgens u meer belang bij hebben?
9. **Afsluiting**
  ▪ Dit is het einde van het interview
  ▪ Zoals aangegeven bij de start van het interview, zullen we u binnen een week een schriftelijke samenvatting met de belangrijkste punten sturen van dit interview. Deze samenvatting kunt u dan doorlezen en vervolgens willen wij u vragen of u kunt kijken of u nog aanpassingen of aanvullingen heeft. Naar welk e-mailadres kan dit gestuurd worden?
  **▪ Bedank de respondent voor de medewerking.**

**Topiclijst semi-gestructureerd interview**

**PATIËNTEN/MANTELZORG**

1. **Introductie:**
  - *Voorstellen interviewer.*
  - *Korte uitleg over de studie en de vragen die aan bod komen.*
  Om geriatrische revalidatie toegankelijk en betaalbaar te houden wordt er door Valkenhof samen met de Universiteit Maastricht onderzoek gedaan naar nieuwe manieren om deze zorg vorm te geven. Wij willen graag onderzoeken of telerevalidatie een zinvolle aanvulling kan zijn voor mensen die thuis wonen en ambulante geriatrische revalidatie ontvangen. Onder ambulante GRZ verstaan we in deze studie revalidatiezorg die in de thuissituatie of poliklinisch (dus als dagbehandeling) wordt aangeboden.
  - *De term ‘telerevalidatie’ uitleggen, navragen of het duidelijk is.*
  In dit onderzoek bedoelen we met telerevalidatie revalidatiezorg die op afstand wordt aangeboden met behulp van technologie. Een voorbeeld hiervan is een behandelaar die een client begeleidt bij het thuis doen van oefeningen, via videobellen of telefonisch contact. Is dit duidelijk voor u?
  - *Toestemming vragen voor deelname en opname van het gesprek*
  - *Uitleg over wat er met de gegevens wordt gedaan*
  Graag wil ik even toelichten wat er met de gegevens van dit interview gebeurt. Het interview wordt opgenomen met behulp van een telefoon. Is het akkoord voor u dat wij het gesprek opnemen? De opname van het interview wordt uitgeschreven en daarna geanalyseerd. Vervolgens wordt een samenvatting van het gesprek gemaakt die we u toesturen om na te gaan of we uw antwoorden goed hebben geïnterpreteerd. Dit kan via email als u dat heeft of via de post of via meegeven met behandelaar. Wat heeft u voorkeur? *(zorg dat je aan het eind van het gesprek de contactgegevens vraagt)*
  Als u nog aanpassingen of aanvullingen hebt kunt u dat aan ons doorgeven binnen 5 werkdagen via een email/antwoordenvelop. Uw gegevens worden vervolgens geanonimiseerd verwerkt. Dit betekent dat in de uiteindelijke beschrijving van de resultaten uw naam niet genoemd wordt en de gegevens niet herleidbaar zullen zijn, naar u als persoon.
  - *Aangeven dat het gesprek naar verwachting 30 min duurt en men altijd tussendoor kan aangeven om te pauzeren.*
  Tijdens dit interview gaan wij u vragen stellen over uw eigen ervaring met het revalidatietraject binnen Valkenhof en uw mening over verschillende nieuwe vormen van revalidatie zoals revalidatie in de thuissituatie en het gebruik van telerevalidatie (bijv. behandeling via videobellen of het meekrijgen van oefeningen op een app voor uw telefoon of tablet).
2. **Achtergrondkenmerken**
  - Bevragen/noteren kenmerken van de respondent. Dit zijn:
    – Geslacht
    – Leeftijd
    – Wat is uw hoogst genoten opleiding?
3. **Uw revalidatiebehandeling**
  - Om welke reden moest u revalideren?
  - Hoe lang bent u opgenomen geweest in het revalidatiecentrum van Valkenhof?
  - Heeft u na uw opname in het revalidatiecentrum ook nog ambulante revalidatie van de behandelaren van Valkenhof ontvangen? *Hiermee bedoelen we een behandeling thuis of poliklinisch (dagbehandeling) door de behandelaren uit het revalidatiecentrum?*
    – Zo ja:
      ▪ Was dit in de vorm van dagbehandeling of behandeling bij u thuis of gecombineerd?
      ▪ Wat vond u van deze behandeling? Waren er dingen die u goed of minder goed vond gaan? Wat waren de redenen dat u het minder goed vond gaan?
    – Zo nee:
      ▪ Wat had u ervan gevonden als u door de behandelaren van Valkenhof ook een tijdje thuis was behandeld? Of via dagbehandeling?
      ▪ Wat zouden hiervan mogelijk de voordelen zijn geweest voor u? *(maak onderscheid tussen thuisbehandeling en poliklinisch)*
      ▪ Wat zouden hiervan mogelijk de nadelen zijn geweest voor u? *(maak onderscheid tussen thuisbehandeling en poliklinisch)*
  - Heeft u tijdens uw revalidatiebehandeling bij Valkenhof wel eens gebruik gemaakt van telerevalidatie? Bijv. van videobellen met uw therapeut, of het gebruik van een revalidatieapp met oefeningen, of ander digitaal contact?
    – Zo ja, waar heeft u gebruik van gemaakt en wat vond u ervan?
  - Heeft u na afloop van de revalidatie bij Valkenhof (dus na de opname en eventueel ambulante revalidatie) vervolgzorg ontvangen van behandelaren in de eerste lijn? Bijvoorbeeld van een fysiotherepeut of ergotherapeut?
    – Zo ja, welke behandeling heeft u ontvangen?
    – Hoe lang duurde deze behandeling (of is deze nog steeds bezig)?
    – Wat vond/vind u van deze behandeling? Zijn er dingen die u goed of minder goed vond gaan?
    – Heeft u bij deze behandeling wel eens gebruik gemaakt telerevalidatie? Bijv. van videobellen met uw therapeut, of het gebruik van een revalidatieapp met oefeningen, of ander digitaal contact?
4. **De mogelijke rol van telerevalidatie**
  *Indien de patiënt bij vraag 2 heeft benoemt dat er dingen minder goed gingen tijdens de ambulante revalidatie en/of van de eerstelijns behandeling na ontslag. Stel dan onderstaande vragen. Zo nee ga door naar vraag 4:*
  - U noemde bij de eerdere vragen dat de volgende dingen [*noem de genoemde dingen*] niet zo goed gingen.
    – Hoe zouden deze dingen volgens u verbeterd kunnen worden?
    – In hoeverre zou telerevalidatie hier ook nog bij kunnen helpen? *(leg indien nodig nogmaals uit wat je bedoelt -> bijv. videobellen met uw therapeut, zelfstandig oefenen via een app)*
      ▪ Zo ja, hoe zou dit er dan uit moeten zien volgens u? Benoem alle aspecten die voor u hierbij belangrijk zijn.
      ▪ Zo nee wat is de reden dat u denkt dat telerevalidatie hier niet bij kan helpen?
5. **Het gebruik van telerevalidatie in de ambulante GRZ**
  - *Deze vraag stellen bij mensen die geen gebruik hebben gemaakt van telerevalidatie:*
    Had u gebuik willen maken van telerevalidatie bij uw revalidatiebehandeling als dit mogelijk zou zijn geweest? *(leg indien nodig nogmaals uit wat je bedoeld -> bijv. videobellen met uw therapeut, zelfstandig oefenen via een app)*
    – Indien ja, kunt u dit toelichten:
      ▪ Wanneer zou u het hebben willen gebruiken? Tijdens opname? Bij poliklinische revalidatiebehandeling? Bij behandeling thuis door therapeuten Valkenhof? Bij de eerstelijns behandeling?
      ▪ Van welke vorm(en) van telerevalidatie had u gebruik willen maken?
    – Indien nee kunt u toelichten waarom u geen gebruik van telerevalidatie had willen maken?
  - Wat zouden volgens u de voordelen kunnen zijn van het gebruik van telerevalidatie bij uw revalidatie behandeling?
    – Voordelen voor u zelf? (niet hoeven reizen, zelfstandig kunnen oefenen, etc.)
    – Voordelen voor uw naasten?
    – Andere voordelen (bijvoorbeeld voor de zorgverleners)
  - Wat zouden volgens u de nadelen kunnen zijn van het gebruik van telerevalidatie bij uw revalidatie behandeling?
    – Nadelen voor u zelf? *(geen persoonlijk contact, te ingewikkeld om met technologie te werken, bang het verkeerd te doen of te vallen, etc.)*
    – Nadelen voor uw naasten?
    – Andere nadelen (bijvoorbeeld voor de zorgverleners)
  - Wat kunnen volgens u de nadelen zijn van telerevalidatie in een ambulante revalidatie traject? (*geen mobiele telefoon/tablet, geen idee hoe deze te gebruiken, bang zonder begeleiding tijdens oefeningen, minder persoonlijk*)
  - Welke dingen kunnen volgens u het gebruik van telerevalidatie volgens u belemmeren? *(bijv. geen goede aparatuur hebben, geen wifi, te moeilijk etc.)*
  - Welke dingen kunnen volgens u het gebruik van telerevalidatie makkelijker maken?
  - Wat is er volgens u nodig nodig om telerevalidatie te kunnen gebruiken als patiënt?
    – Beschikbare materialen en voorzieningen?
    – Bijvoorbeeld technische ondersteuning voor het internet of het gebruik van een tablet?
    – Educatie patiënten en naasten
    – Vormgeving van de te gebruiken software?
    – Organisatie van de zorg?
    – Ondersteuning door naasten?
    – Overig?
  - Als aan deze voorwaarden zou kunnen worden voldaan, zou u dan gebruik willen maken van telerevalidatie in de toekomst?
6. **Afsluiting**
  - Dit is het einde van het interview
  Zoals aangegeven bij de start van het interview, zullen we u binnen een week een schriftelijke samenvatting met de belangrijkste punten sturen van dit interview, zodat u dit kunt doorlezen en vervolgens willen wij u vragen of u kunt kijken of u nog aanpassingen of aanvullingen heeft.
  Maak afspraken over hoe de samenvatting bij de patient komt en weer terug naar jullie. Wissel contactgegevens uit indien nodig.
  – **Bedank de respondent voor de medewerking.**

## Appendix B Full Interview Scheme (Translated)

**Topic list semi-structured interview**

**CARE PROFESSIONALS/OTHER DISCIPLINES**

1. **Introduction:**
  - *Introducing the interviewer.*
  - *Brief explanation of the study and the questions addressed.*
  To keep geriatric rehabilitation accessible and affordable, Stichting Valkenhof, together with Maastricht University, is researching new ways to shape this type of care. We would like to investigate whether telerehabilitation can be a useful addition for people who live at home and receive home-based geriatric rehabilitation. In this study, home-based GR means rehabilitation care offered in the home or home-based setting.
  - *Explain the term ‘telerehabilitation’, inquire if it is clear.*
  In this study, by telerehabilitation we mean rehabilitation care offered remotely using technology. An example would be a practitioner assisting a client in doing exercises at home, via video calling or telephone contact. Is this clear to you?
  - *Requesting permission to participate and record the interview*
  - *Explaining what will be done with the data*
  Let me briefly explain what happens with the data from this interview. The interview will be recorded with a telephone. Is it okay for us to record the interview? The recording of the interview is written out and analysed. A summary of the interview is then made which we will send to you to check whether we have interpreted your answers correctly. If you have any adjustments or additions, you can let us know within 5 working days. Your data will then be processed anonymously. This means that in the final description of the results, your name will not be mentioned and the data will not be traceable to you as a person.
  - *Indicate that the interview is expected to last 30 min and one can always indicate to take a break in between.*
  During this interview, we are going to ask you questions about the current application of home-based geriatric rehabilitation and the current use of telerehabilitation within Stichting Valkenhof. We are going to ask you first about your opinion on the organisation of home-based GR within Stichting Valkenhof. We are then going to ask you about the possible role and application of telerehabilitation within home-based GR. And finally, we will ask you questions about who has or could have an interest in applying telerehabilitation in home-based GR (identifying stakeholders).
2. **Background characteristics:**
  - Questioning/noting characteristics of the respondent. These are:
    – Gender
    – Age
    – Profession
    – Working experience (years)
3. **The current use of home-based GR and telerehabilitation within Stichting Valkenhof**
  - To what extent does Stichting Valkenhof offer home-based GR? In what form is it offered (outpatient or at the patient's home?)
  - To what extent are you personally involved in the implementation of home-based GR?
    – In what form do you offer home-based GR (outpatient or at the patient's home?)
    – *If the person is not involved in home-based GR, ask*:
  To what extent are you involved in primary care of GR patients discharged home?
  - To what extent is telerehabilitation used within your organisation?
    – Where is this applied? GR-clinical? Home-based GR? GR home treatment in outpatient GR? In primary care?
    – What forms of telerehabilitation are used?
  - To what extent do you use telerehabilitation yourself?
    – Where do you use this? GR-clinical? Home-based GR? GR home treatment in outpatient GR? In primary care?
    – What forms of telerehabilitation do you use?
      ▪ Remote therapy via video call?
      ▪ Using rehabilitation apps together with the patient, e.g. for doing exercises?
      ▪ Using motion sensors and coaching/monitoring the patient based on them?
      ▪ Other, namely.
4. **Your opinion on home-based GR**
  - What is your opinion on home-based GR?
    – What do you think is going well? Can you cite any examples?
    – What do you think is going less well? Can you cite any examples?
    – Can you indicate the causes of the things you feel are going less well?
    – What things do you think could be improved in home-based GR?
  - *If one has indicated that no home-based GR is offered at Stichting Valkenhof, ask the questions below*:
    – Why is home-based GR not applied at Stichting Valkenhof?
    – How do you feel about the lack of home-based GR?
    – To what extent do you think home-based GR should be implemented? What form should it take (outpatient, treatment at home)?
5. **The possible role of telerehabilitation within home-based GR**
  - *If people indicated in question 4 that there are areas for improvement in home-based GR, ask the questions below:*
  You indicate that the following things could be improved in home-based GR: *[list here the things mentioned in question 4]*. Do you think telerehabilitation could play a role in this?
  – If so, could you please explain? In your opinion, what should this look like? What is important here?
  – If not, do you perhaps see other opportunities to improve the home-based rehabilitation process with the use of telerehabilitation?
    ▪ If so, what do you think this should look like? Name all aspects that are important to you in this regard.
    ▪ If not, what is the reason that you do not see opportunities to improve the rehabilitation process with the use of telerehabilitation?
  - *If people indicated in question 4 that home-based GR is provided but there are no areas for improvement, or that no home-based GR is provided, then ask the questions below:*
  Do you see any opportunities to improve the home-based rehabilitation process (in general or within Stichting Valkenhof) with the use of telerehabilitation?
  – If so, what do you think this should look like? Name all aspects that are important to you in this regard.
  – If not, what is the reason that you do not see opportunities to improve the rehabilitation process with the use of telerehabilitation?
6. **Potential added value and weaknesses of telerehabilitation within home-based GR**
  - What do you think is the added value of telerehabilitation in a home-based rehabilitation programme?
    – For the patient?
    – For loved ones/informal caregivers?
    – For care providers?
    – For the care organisation?
    – For effectiveness/efficiency of care?
    – For other matters, namely:
  - To what extent do you think there are weaknesses to using telerehabilitation in a home-based rehabilitation programme?
    – For the patient?
    – For loved ones/informal caregivers?
    – For care providers?
    – For the care organisation?
    – For effectiveness/efficiency of care?
    – For other matters, namely:
7. **Factors that may influence application of telerehabilitation**
  - What factors do you think could facilitate the use of telerehabilitation in a home-based rehabilitation programme?
  - What factors do you think might inhibit the use of telerehabilitation in a home-based rehabilitation programme?
  - What do you think is needed to properly implement telerehabilitation in home-based GR (within Stichting Valkenhof)? Both for patients, informal caregivers, care professionals and the care organisation?
    – Available materials and facilities?
    – Staff training
    – Educating patients and informal caregivers
    – Design of the software to be used?
    – Organisation of care?
    – Patient support from informal caregivers?
    – Insurance regulations for telerehabilitation treatment? Adjust current regulations?
    – Availability of care pathways or guidelines?
    – Other?
8. **The interest of different parties (stakeholders) in applying telerehabilitation in home-based GR?**
  *Certain people may have varying degrees of interest in applying telerehabilitation in home-based GR. Therefore, we would now like to look at who may have an interest in applying telerehabilitation in home-based GR.*
  - Who do you think has an interest in applying telerehabilitation in home-based GR?
  - Who has the most interest? Do certain people/parties have more interest in it than others?
    – If so, who would be more important?
    – What are the reasons you think these persons/parties have more interest in it?
9. **Closing**
  ▪ This is the end of the interview
  ▪ As indicated at the start of the interview, we will send you a written summary of the main points of this interview within a week. You can then read through this summary and then we would like to ask you to see if you have any adjustments or additions. To which e-mail address can this be sent?

**Thank the respondent for their cooperation.**

**Topic list semi-structured interview**

**PATIENTS/INFORMAL CAREGIVER**

1. **Introduction:**
  - *Introducing the interviewer.*
  - *Brief explanation of the study and the questions addressed.*
  To keep geriatric rehabilitation accessible and affordable, Stichting Valkenhof, together with Maastricht University, is researching new ways to shape this care. We would like to investigate whether telerehabilitation can be a useful addition for people who live at home and receive home-based geriatric rehabilitation. In this study, home-based GR means rehabilitation care offered in the home situation or outpatient (i.e. as day treatment).
  - *Explain the term ‘telerehabilitation’, inquire if it is clear.*
  In this study, by telerehabilitation we mean rehabilitation care offered remotely using technology. An example would be a practitioner assisting a client in doing exercises at home, via video calling or telephone contact. Is this clear to you?
  - *Requesting permission to participate and record the interview*
  - *Explaining what will be done with the data*
  Let me briefly explain what happens to the data from this interview. The interview will be recorded using a telephone. Is it okay for us to record the interview? The recording of the interview is written out and then analysed. A summary of the interview will then be sent to you to check whether we have interpreted your answers correctly. This can be done via email if you have it, by post or by passing it along with your care professional. Which do you prefer? *(make sure you ask for contact details at the end of the interview)*
  If you have any adjustments or additions, please let us know within 5 working days via email/answering by your care professional. Your data will then be processed anonymously. This means that in the final description of the results, your name will not be mentioned and the data will not be traceable, to you as a person.
  - *Indicate that the interview is expected to last 30 min and one can always indicate to take a break in between.*
  During this interview, we will ask you questions about your own experience of the rehabilitation process within Stichting Valkenhof and your opinion on various new forms of rehabilitation such as rehabilitation in the home situation and the use of telerehabilitation (e.g. treatment via video calls or taking exercises with you on an app for your phone or tablet).
2. **Background characteristics**
  - Questioning/noting characteristics of the respondent. These are:
    – Gender
    – Age
    – What is your highest level of education?
3. **Your rehabilitation treatment**
  - For what reason did you need rehabilitation?
  - How long were you admitted to Stichting Valkenhof rehabilitation center?
  - After your admission to the rehabilitation center, did you also receive home-based rehabilitation from by Stichting Valkenhof? *By this we mean treatment at home or outpatient (day treatment) by the rehabilitation centre's practitioners?*
    – If so:
      ▪ Was this in the form of day care or treatment at your home or combined?
      ▪ What did you think of this treatment? Were there things you thought went well or less well? What were the reasons you thought it went less well?
    – If not:
      ▪ How would you have felt if Stichting Valkenhof's therapists had also treated you at home for a while? Or via day treatment?
      ▪ What would possibly have been the benefits of this for you? *(distinguish between home treatment and outpatient)*
      ▪ What would possibly have been the disadvantages of this for you? *(distinguish between home treatment and outpatient)*
  - Have you ever used telerehabilitation during your rehabilitation treatment at Valkenhof?
  E.g. video calling with your therapist, or using a rehabilitation app with exercises, or other contact digitally?
  – If so, what did you use and what did you think of it?
  - Did you receive follow-up care from primary care practitioners after your rehabilitation at Valkenhof (i.e. after dismissal from the rehabilitation care ward)? For example, from a physiotherapist or occupational therapist?
    – If yes, what treatment did you receive?
    – How long did this treatment last (or is it still in progress)?
    – What did you think/feel about this treatment? Are there things you thought went well or less well?
    – Have you ever used telerehabilitation in this treatment? E.g. video calling with your therapist, or using a rehabilitation app with exercises, or other contact digitally?
4. **The potential role of telerehabilitation**
  *If, in question 2, the patient has mentioned that things did not go as well during outpatient rehabilitation and/or from primary care treatment after discharge, then ask the questions below. If not, proceed to question 4:*
  - You mentioned in the earlier questions that the following things [*name the things mentioned*] did not go so well.
    – How do you think these things could be improved?
    – To what extent could telerehabilitation also help with this? *(If necessary, explain again what you mean -> e.g. video calling with your therapist, practising independently via an app)*
      ▪ If so, what do you think this should look like? Name all aspects that are important to you in this regard.
      ▪ If not, what is the reason you think telerehabilitation cannot help with this?
5. **The use of telerehabilitation in outpatient GR**
  - *Ask this question of people who have not used telerehabilitation:*
    Would you have used telerehabilitation in your rehabilitation treatment if this had been possible? *(if necessary, explain again what you mean -> e.g. video calling your therapist, practising independently via an app)*
    – If yes, please explain:
      ▪ When would you have wanted to use it? During admission? During outpatient rehabilitation treatment? During treatment at home by therapists from Valkenhof? During primary care treatment?
      ▪ What form(s) of telerehabilitation would you have liked to make use of?
    – If not, can you explain why you would not have wanted to use telerehabilitation?
  - What do you think could be the benefits of using telerehabilitation in your rehabilitation treatment?
    – Benefits for yourself? (Not having to travel, being able to practise independently, etc.)
    – Benefits for your informal caregiver(s)?
    – Other benefits?
  - What do you think might be the disadvantages of using telerehabilitation in your rehabilitation treatment?
    – Disadvantages for yourself? *(No personal contact, too complicated to work with technology, afraid of doing it wrong or falling down, etc.)*
    – Drawbacks for your informal caregiver(s)?
    – Other disadvantages?
  - What do you think might be the weaknesses of telerehabilitation in home-based rehabilitation? (*no access to a mobile phone/tablet, no idea how to use it, scared without guidance during exercises, less personal*)
  - In your opinion, what things can hinder the use of telerehabilitation? *(e.g. not having good equipment, no wifi, too difficult etc.)*
  - What things do you think could make using telerehabilitation easier?
  - What do you think is needed to use telerehabilitation as a patient?
    – Available materials and facilities?
    – For example, technical support for the internet or using a tablet?
    – Educating patients and informal caregiver
    – Design of the software to be used?
    – Organisation of care?
    – Support from informal caregiver?
    – Other?
  - If these conditions could be met, would you want to use telerehabilitation in the future?
6. **Closing**
  - This is the end of the interview
  As indicated at the start of the interview, we will send you a written summary with the main points of this interview within a week for you to read through and then we would like to ask you to see if you have any adjustments or additions.
  Agree how the summary will get to the patient and back to you. Exchange contact details if necessary.
  – **Thank the respondent for their cooperation.**

## Appendix C Data on the Current Application and Experience with Home-based GR and Telerehabilitation (within Stichting Valkenhof)

**Table 4:** Current Application of Home-based GR with Strengths and Weaknesses

Main themes/sub-themes	Definition of theme	Codes^a^	CP^b^	P/IC^c^
**Current state of home-based GR (within Stichting Valkenhof)**				
- **Unclear reimbursement system**	- The reimbursement structure of homebased GRwas unclear.	**4**	**4**	**-**
- **Regulatory difficulties**	- Current regulations require certain conditions that impede the use of homebased GR.	**3**	**3**	**-**
- **Frail population**	- Patients may lack the physical and/or mental capacity to return home safely for GR.	**2**	**2**	**1**
- **Merit of home-based GR**	- Home-based GR might be more adequate after a (short) inpatient stay beforehand.	**3**	**3**	**-**
**Strengths of home-based GR**				
- **Improved independent functioning**	- Practising skills in the home environment might be more beneficial to improve independent functioning.	**6**	**5**	**1**
- **Improved patient wellbeing**	- Patients might feel more comfortable rehabilitating in their own environment.	**4**	**-**	**4**
- **Improved motivation**	- Patients can be more motivated at home.	**1**	**-**	**1**
- **Multidisciplinary work advantages**	- The multidisciplinary collaboration and communication is of high quality.	**10**	**10**	**-**
**Weaknesses of home-based GR (barrier for adoption)**				
- **Unplanned care moments**	- Unplanned care is difficult to provide at home.	**3**	**3**	**-**
- **Presence of an informal caregiver**	- Support of an informal caregiver may be required for a successful home-based GR trajectory.	**5**	**5**	**-**
- **Transition of care difficulties**	- The transition of inpatient to home-based care might be overwhelming and frightening.	**7**	**7**	**-**
- **Travel time burden**	- For either the patient or healthcare professional, the travel time can be a burden.	**8**	**6**	**2**
- **Unsuitable home environment**	- Rehabilitation at home might not be suitable for patient and/or healthcare professional	**2**	**1**	**1**

*Note*. *CP*, care professional; *P*, patient; *IC*, informal caregiver*; GR*, geriatric rehabilitation;

a The total number of times a code was mentioned in all interviews;

b The number of different therapists that mentioned a code;

c The number of patients or informal caregiver that mentioned a code

For data on the current application of home-based GR, the main themes, identified sub-themes and their definitions are provided in table 4. The current application of home-based GR was categorized in the strengths and the weaknesses of home-based GR.

Overall, the care professionals indicated that home-based GR was only rarely applied within Stichting Valkenhof. While it was not evidently clear why this was the case, the care professionals argued it was due to the current GR regulations, in addition to hesitations for providing home-based care when dealing with the frail target population. Hence, all three included patients received home-based or outpatient *primary care* following their inpatient GR.

Quote: *“I have been working here for a long time now, so I've provided home-based rehabilitation in the past. I think it is part of the job, it is a loss that we do not offer it anymore. I think that's due to certain rules, as well as the financing which are unclear, in that it is not entirely clear whether we will receive money for this.”* (CP 7)

Quote: *“Mainly, it is the frailty of the patients and the home living environment which are often not suitable, certainly in the early stages of rehabilitation. Maybe if someone has already been rehabilitated for 6 weeks then it is possible, but the living environment is also still a problem.”* (CP19)

Despite the challenges with the current GR regulations or patient suitability, multiple care professionals indicated their belief that, following an initial inpatient stay, the rehabilitation process should be followed-up by a home-based trajectory. Care professional 10 explained that:

Quote: *“I think we should grow in this; so, I am a proponent of implementing more home-based GR and preferably if it's used in conjunction with an inpatient stay, with afterwards coming home to continue.”*

### Strengths of Home-based GR

All identified sub-themes regarding the strengths of home-based GR are provided in Table 4. The majority of the care professionals indicated that multidisciplinary collaboration and communication is one of the primary strengths of home-based GR. There is a variety of situations in which this is applicable, for example when coordinating treatment appointments, or when multiple care professionals jointly treat a particular patient problem. The following statements illustrate:

Quote: *“[…] so, when somebody wants to reduce tube feeding and suffers from swallowing problems, we together can examine how we want to treat this patient as best as possible”.* (CP 12)

Quote: *“We always agree with colleagues on what days are you going, so that in the end we distribute it over the whole week and make sure the burden of the patient is acceptable.”* (CP 10)

The ability to practise skills and other exercises with a patient in the home environment was seen as a strength by several care professionals, as well as one patient (P 3). This patient also concluded that the home-based care exceeded expectations, in particular to function independently. This patient felt more comfortable at home, as opposed to a continuation of the inpatient treatment. The home environment felt pleasant and familiar, and the informal caregiver in particular was seen as a stimulating factor. Additionally, this patient appreciated the fact that he did not need to travel to care facility.

Quote: *“This really was a nice advantage (…of home-based GR); I would not know how I would go to the care facility and what I would need to arrange for that and what to organize”. […] it (…home-based GR) really has helped me with my rehabilitation process and contributed to my independent functioning.”* (P 3)

### Weaknesses of Home-based GR

All identified sub-themes regarding the weaknesses of home-based GR are provided in table 4. Multiple care professionals indicated that the presence of an informal caregiver was considered as an important prerequisite for a successful home-based GR trajectory. This is partly due to the fact that informal caregivers may be required to deal with unplanned care, which is one of the most common reasons for denying a home-based GR trajectory. Furthermore, several care professionals described how the transition from inpatient to home-based GR could negatively impact the patient. During an inpatient stay, there is a highly structured daily schedule. In contrast, home-based GR requires patients to operate more independently. There is a risk in having the transition take place without careful consideration. One care professional described this situation in the following:

Quote: *“Here, we provide everything. You have a whole schedule and team composed of therapists, care professionals, supporting staff, entertainment personnel etc. and this is for example until Thursday, and on Friday you are home again, and everything is gone.”* (CP 18)

### Experience with Telerehabilitation (in Relation to GR)

The interviewees were asked on the current (or previous) use of any kind of telerehabilitation during (home-based) GR or primary care of recently discharged GR patients. Of all care professionals, only one dietician indicated to occasionally use an app as a food diary.

Quote: *“We sometimes use an app as a food diary. […] Recently, a new app has also been launched, especially for keeping track of protein intake. We are working on to getting started with that app so that people can easily monitor their protein intake themselves.”* (CP 11)

When asked if there was any form of digital communication, not necessarily with an app or video contact, the speech and occupational therapists acknowledged this occurrence.

Quote: *“The only thing we do sometimes is practice speech over the phone. We do that when things are getting better, or we call to see how someone is doing in terms of swallowing, for example. Yes, we do things by phone, but not really in terms of video calling or things like that.”* (CP 9)
